# Integrative Omic Analysis Reveals the Dynamic Change in Phenylpropanoid Metabolism in *Morus alba* under Different Stress

**DOI:** 10.3390/plants12183265

**Published:** 2023-09-14

**Authors:** Yaohan Li, Shengzhi Liu, Di Zhang, Amin Liu, Wei Zhu, Jianbin Zhang, Bingxian Yang

**Affiliations:** 1 College of Biomedical Engineering & Instrument Science, Zhejiang University, Hangzhou 310027, China; 12115001@zju.edu.cn (Y.L.); bylzs8410@163.com (S.L.); 12115002@zju.edu.cn (A.L.); 2 Key Laboratory for Molecular Medicine and Chinese Medicine Preparations, Institute of Basic Medicine and Cancer, Chinese Academy of Sciences, Hangzhou 310022, China; zhangdi202102@163.com; 3 Changshu Qiushi Technology Co., Ltd., Suzhou 215500, China; rutin@zju.edu.cn; 4 College of Life Sciences and Medicine, Zhejiang Sci-Tech University, Hangzhou 310018, China

**Keywords:** phenylpropanoid metabolism, *Morus* leaves, proteomics, metabolomics, stress

## Abstract

*Morus alba* is used as a traditional Chinese medicine due to its various biological activities. Phenylpropanoid metabolism is one of the most important pathways in *Morus alba* to produce secondary metabolites and response to stress. From the general phenylpropanoid pathway, there are two metabolic branches in *M. alba*, including flavonoid and lignin biosynthesis, which also play roles in response to stress. However, the dynamic changes between flavonoid and lignin biosynthesis under *Botrytis cinerea* infection and UV-B stress in *M. alba* were unclear. To explore the different regulation mode of flavonoid and lignin biosynthesis in *M. alba* leaves’ response to biotic and abiotic stress, a combined proteomic and metabolomic study of *M. alba* leaves under UV-B stress and *B. cinerea* infection was performed. The results showed that most of the proteins involved in the lignin and flavonoid biosynthesis pathway were increased under either UV-B stress or *B. cinerea* infection in *M. alba*. This was also confirmed by enzyme assays and metabolomics analysis. Additionally, the abundance of proteins involved in the biosynthesis of jasmonic acid was increased after *B. cinerea* infection. This suggests that both flavonoid and lignin biosynthesis participate in the responses to abiotic and biotic stress in *M. alba*, but they might be regulated by different hormone signaling.

## 1. Introduction

Phenylpropanoid metabolism is one of the most important metabolisms in plants, yielding more than 8000 metabolites which contribute to plant development and plant-environment interplay [1]. Flavonoid biosynthesis is an important branch of phenylpropanoid metabolism encompassing over 6000 compounds, which gives rise to the largest class of polyphenolic metabolites [2,3]. Flavonoids play a very important role in the survival of plants under diverse biotic and abiotic stresses. In angiosperms, the UV RESISTANCE LOCUS8 (UVR8) photoreceptor coordinates UV-B responses and induces the biosynthesis of protective flavonoids in defense of UV-B radiation [4]. The accumulation of flavonoids induced by the bHLH transcription factor (TF) enhances salt and drought tolerance in transgenic *Arabidopsis thaliana* [5]. There was also evidence to support that flavonoid metabolism improved adaptation to biotic stress in blackberry and *Arabidopsis* [6,7].

Lignin biosynthesis is another important branch of phenylpropanoid metabolism. Lignin is a complex biomacromolecular polymer that usually surrounds the polysaccharide components of plant cell walls and provides structural support, giving rigidity and strength to stems [8]. The activation of lignin biosynthesis also serves as a strategy for stress resistance. Promoted lignin and proanthocyanidin biosynthesis in the roots of *Populus ussuriensis* resulted in enhanced tolerance to drought stress by means of anti-oxidation and mechanical supporting [9]. In addition, lignin can reduce the water penetration and transpiration of the plant cell wall, which helps to maintain cell osmotic balance and protect membrane integrity under the osmotic stress caused by extreme drought and high salt stress [10]. Moreover, the accumulation of lignin can provide a basic barrier against pathogen spread and reduce the infiltration of fungal enzymes and toxins into plant cell walls [11].

Lignin biosynthesis and flavonoid biosynthesis share the same precursor and early steps. Both of them could contribute to the tolerance of biotic and abiotic stress. However, a preference for lignin biosynthesis and the suppression of flavonoid biosynthesis has been reported in the studies of some species under biotic stress. The accumulation of flavonols induced by UV-B was attenuated by the concurrent application of the bacterial elicitor flg22 (simulating biotic stress), which correlated with the strong suppression of the flavonol biosynthesis genes [12]. The suppression of flavonoid accumulation was also observed in onion plants infected by *Botrytis allii* [13]. Since the phenylpropanoids’ metabolism has the same precursor, the suppression of flavonoid biosynthesis allows more resources for the biosynthesis of lignin and other phytoalexins. On the other hand, the suppression of flavonoid synthesis might have negative effects on the pathogen defense because flavonoid can contribute to warding off the virulent pathogen [6]. Although this suppression has been observed in some species, whether this phenomenon is common in other species needs more evidence.

*Morus alba* is widely cultivated because its leaves can be used to feed silkworms [14]. *M. alba* is also used as an herb in traditional Chinese medicine. Secondary metabolites of *M. alba* have been proved to have multiple physiological activities, including antiviral, antibacterial, and anti-inflammatory activities [15,16,17,18,19]. To better illustrate the regulation mode of flavonoid biosynthesis and lignin biosynthesis in *M. alba* leaves under different stresses, we treated *M. alba* leaves using two kinds of stress: *B. cinerea* infection, which is biotic stress, and high-level UV-B radiation, which is abiotic stress. Proteomic analysis was performed, and the results showed that flavonoid biosynthesis and lignin biosynthesis were significantly up-regulated by BC stress and UV-B stress. Although *B. cinerea* infection stress is biotic stress, no suppression of flavonoid biosynthesis has been observed compared with UV-B stress. This was confirmed by enzyme activity and metabolite analysis. After comparing the changes in lignin biosynthesis and flavonoid biosynthesis in the *B. cinerea* infection group (BC) and UV-B radiation group (UVB), the results suggested that resistance to UV-B and BC stress may be achieved through different pathways, but both of them could finally activate the biosynthesis of flavonoid and lignin.

## 2. Results

### 2.1. Overview of Differentially Expressed Proteins in the BC Group and UVB Group

**Figure 1:**
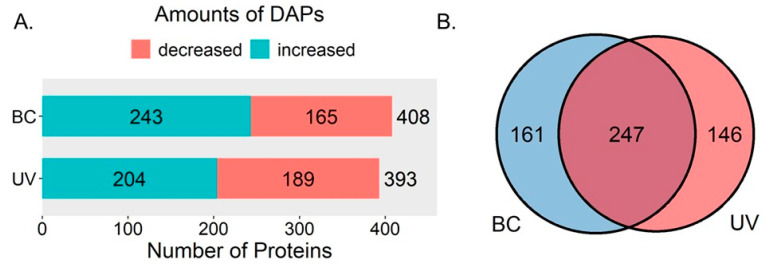
Overviews of DEPs. (**A**) Amounts of increased/decreased DEPs in BC group and UV group. (**B**) Venn diagram analysis of DEPs in UV group and BC group.

A total of 4120 proteins with at least two matched unique peptides were identified and quantified in *M. alba* leaves. In the BC group, 408 differentially expressed proteins (DEPs) were identified, including 165 decreased and 243 increased DEPs. In the UVB group, 393 DEPs were identified. Among them, 189 DEPs were decreased and 204 were increased (Figure 1A). In total, there were 247 common DEPs between the UVB and BC groups, with 146 proteins differentially expressed in the UVB group exclusively and 161 proteins differentially expressed in the BC group exclusively (Figure 1B).

### 2.2. GO Term and KEGG Pathway Enrichment of DEPs in the BC Group

**Figure 2:**
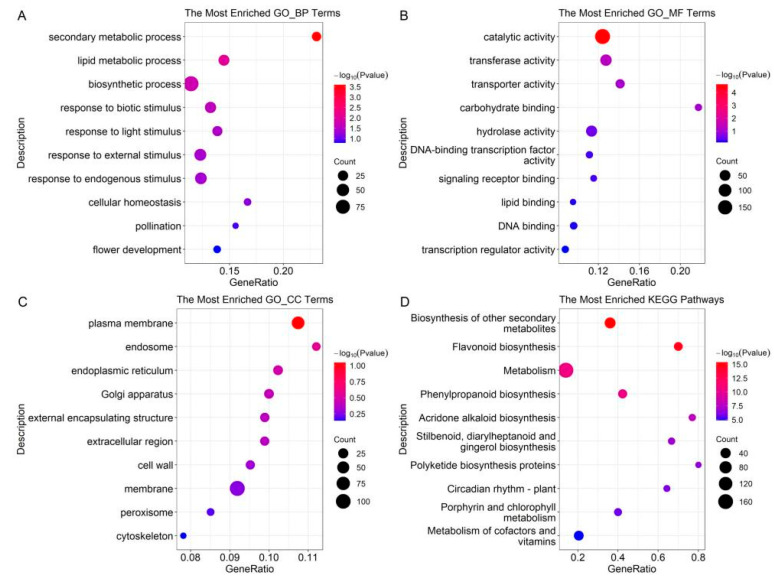
GO and KEGG enrichment of DEPs in BC group. (**A**) Enriched GO biological process terms. (**B**) Enriched GO molecular function terms. (**C**) Enriched GO cellular component terms. (**D**) Enriched KEGG pathways.

The DEPs of the BC group were classified based on GO annotations, and the results showed that secondary metabolic processes, especially phenylpropanoid biosynthesis, were altered under BC infection (Figure 2). In the biological process, the enriched terms included secondary metabolic process, lipid metabolic process, biosynthetic process, response to biotic stimulus, response to light stimulus, etc. In the molecular function, the enriched terms included catalytic activity, transferase activity, transporter activity, carbohydrate binding, hydrolase activity, etc. In the cellular component, the enriched terms included plasma membrane, endosome, endoplasmic reticulum, Golgi apparatus, external encapsulating structure, etc. The enrichment of the KEGG pathways showed that the DEPs of the BC group were involved in various secondary metabolisms, such as flavonoid biosynthesis, phenylpropanoid biosynthesis, acridone alkaloid biosynthesis, etc.

### 2.3. GO Term and KEGG Pathway Enrichment of DEPs in the UVB Group

**Figure 3:**
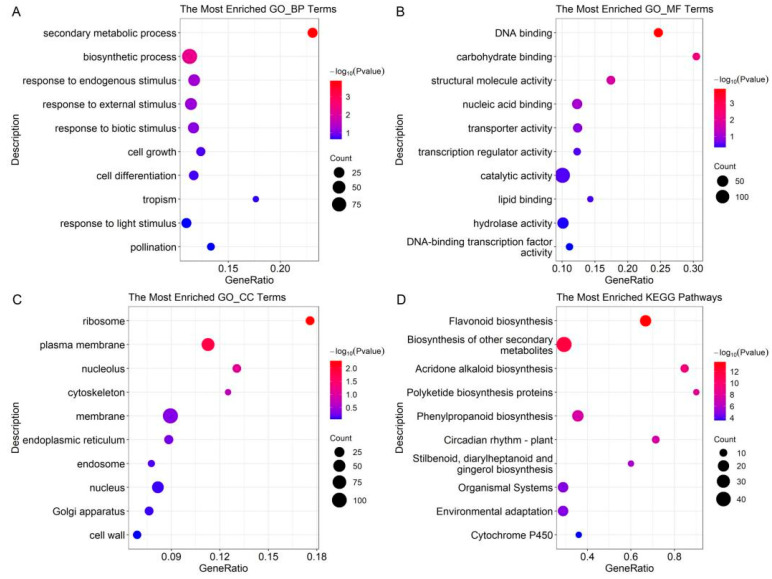
GO and KEGG enrichment of DEPs in UV group. (**A**) Enriched GO biological process terms. (**B**) Enriched GO molecular function terms. (**C**) Enriched GO cellular component terms. (**D**) Enriched KEGG pathways.

The GO term and KEGG pathway enrichment were also performed on the DEPs of the UVB group (Figure 3). It was found that phenylpropanoid biosynthesis and flavonoid biosynthesis were also altered after UV-B treatment. The top 10 enriched GO terms were displayed. In the biological process, the enriched terms included secondary metabolic process, biosynthetic process, response to endogenous, response to external stimulus, response to biotic stimulus, etc. In the molecular function, the enriched terms included DNA binding, carbohydrate binding, structural molecule activity, nucleic acid activity, transporter activity, etc. In the cellular component, the enriched terms included ribosome, plasma membrane, nucleolus, cytoskeleton, membrane, etc. Then, the DEPs of the UVB group were mapped on KEGG pathways. The results showed that DEPs were involved in various secondary metabolisms, including flavonoid biosynthesis, acridone alkaloid biosynthesis, polyketide biosynthesis proteins, phenylpropanoid biosynthesis, etc.

### 2.4. Lignin Biosynthesis Was Enhanced under Either BC or UVB Stress

**Figure 4:**
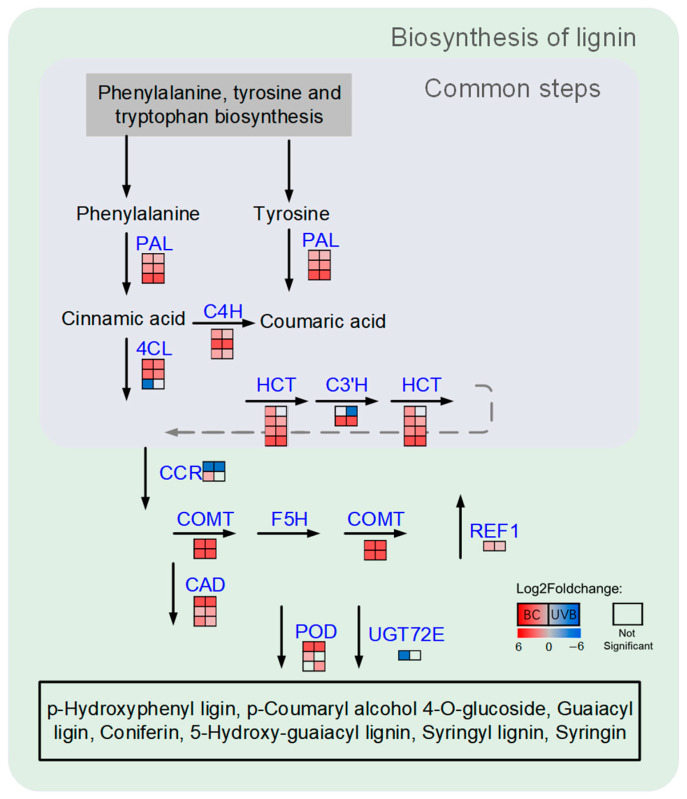
DEPs in lignin biosynthesis. Enzymes involved in biosynthesis of lignin were up-regulated by BC stress and UV stress. Each line of squares represented a protein. Filled color represented Log2Foldchange in proteins. Left column represented Log2Foldchange in BC group, and right column represented Log2Foldchange in UVB group.

**Figure 5:**
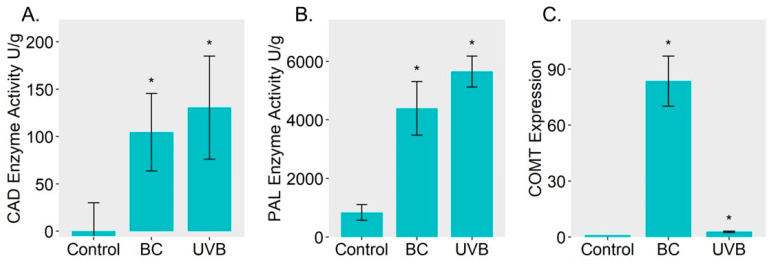
Analysis of physiological changes. (**A**) Enzyme activity of CAD. (**B**) Enzyme activity of PAL. (**C**) Expression level of COMT. Assays on enzyme activity and gene expression further proved the up-regulation of lignin biosynthesis. Asterisks indicate a significant change based on Student’s *t* test (* *p* < 0.05) compared to control group.

The results of functional enrichment showed that either BC treatment or UVB treatment evoked alteration in the biosynthesis of phenylpropanoid. Therefore, the DEPs related to lignin biosynthesis were mapped based on the KEGG database (Figure 4). Most of the DEPs from the BC group or UVB group were increased under stress. For instance, three key enzymes involved in phenylpropanoid biosynthesis, including phenylalanine ammonia-lyase (PAL, EC: 4.3.1.24), 4-coumarate-CoA ligase (4CL, EC: 6.2.1.12), and cinnamate 4-hydroxylase (C4H, EC: 1.14.14.91), were significantly increased in the BC and UVB groups. Moreover, four shikimate O-hydroxycinnamoyl transferase (HCT, EC: 2.3.1.133), two caffeic acid 3-O-methyltransferase (COMT, EC: 2.1.1.68), three cinnamyl-alcohol dehydrogenase (CAD, EC: 1.1.1.195), and a peroxidase (POD, EC: 1.11.1.7), which was involved in the biosynthesis of lignin, were also increased in the BC and UVB groups. This indicates that lignin biosynthesis in *M. alba* might participate in the resistance of stress. Then, enzyme activity assays of PAL and CAD showed that the activity of PAL and CAD was increased under BC infection and UVB stress (Figure 5A,B). The expression level of caffeic acid O-methyltransferase (COMT) was determined by qRT-PCR analysis. It showed that under stress, the expression level of COMT was significantly increased compared to the control group (Figure 5C). Especially for the BC group, the expression level of COMT in the BC group was almost ten times higher than it was in the UVB group.

### 2.5. Flavonoid Biosynthesis Was Enhanced under Either BC or UVB Stress

**Figure 6:**
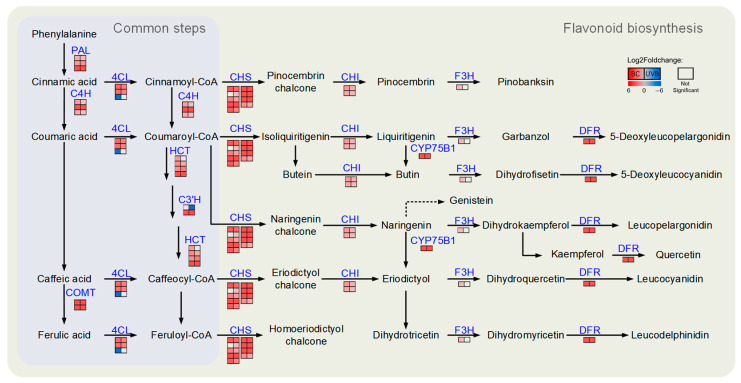
DEPs in flavonoid biosynthesis. Flavonoid biosynthesis was evoked by BC stress and UV stress. Each line of squares represented a protein. Filled color represented Log2Foldchange in proteins. Left column represented Log2Foldchange in BC group, and right column represented Log2Foldchange in UVB group.

**Figure 7:**
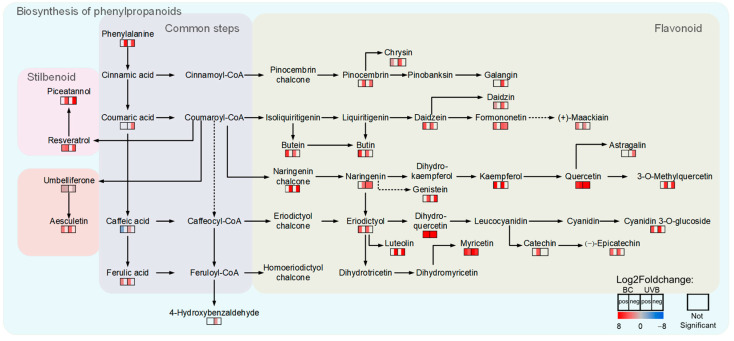
DAMs in flavonoid biosynthesis. Activation of phenylpropanoid biosynthesis was confirmed by non-target metabolomic data. Each line of squares represented a metabolite. Filled color represented Log2Foldchange in metabolites. Left column represented Log2Foldchange in BC group, and right column represented Log2Foldchange in UVB group. Pos represented result in positive ion mass spectrum, and neg represented result in negative ion mass spectrum.

**Figure 8:**
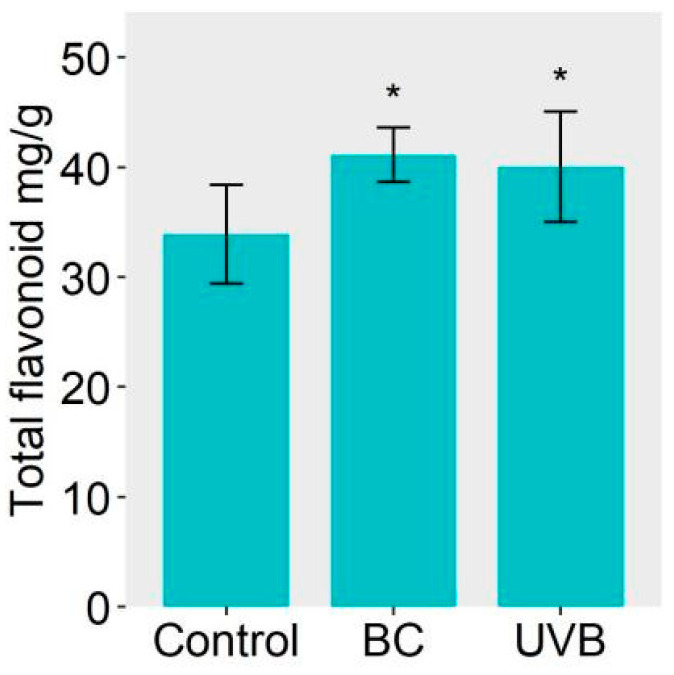
Content of total flavonoid. Both treatments induced the accumulation of total flavonoid, which proved the up-regulation of flavonoid biosynthesis. Asterisks indicate a significant change based on Student’s *t* test (* *p* < 0.05) compared to control group.

The DEPs related to flavonoid biosynthesis from the BC and UVB groups were mapped using the KEGG database. The results showed that the abundance of DEPs involved in flavonoid biosynthesis was increased under either BC or UVB stress (Figure 6). For instance, chalcone synthase (CHS, EC: 2.3.1.74), chalcone isomerase (CHI, EC: 5.5.1.6), flavonoid 3′-monooxygenase (CYP75B1, EC: 1.14.14.82), and bifunctional dihydroflavonol 4-reductase/flavanone 4-reductase (DFR, EC: 1.1.1.234) were significantly increased after BC and UVB treatments. Furthermore, the untargeted metabolomics analysis showed that the contents of chalcones and flavonoids, such as butein, naringenin chalcone, genistein, dihydroquercetin, myricetin, kaempferol, and quercetin, were significantly increased under either BC or UVB stress (Figure 7). This was consistent with the changes in proteomics results. Also, the contents of total flavonoid were significantly increased under either BC or UVB stress (Figure 8)

### 2.6. Proteins Involved in the Biosynthesis of Phenylalanine and Tyrosine Were Increased under BC and UVB Stress

**Figure 9:**
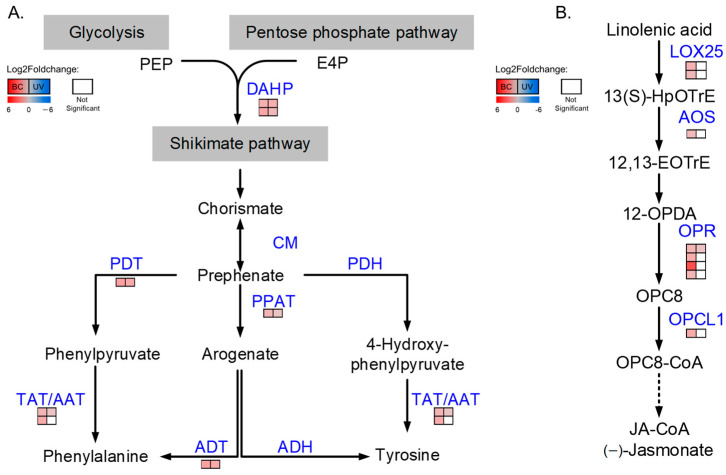
DEPs involved in phenylalanine and tyrosine biosynthesis pathway (**A**) and JA biosynthesis (**B**). Each line of squares represented a protein. Filled color represented Log2Foldchange in proteins. Left column represented Log2Foldchange in BC group, and right column represented Log2Foldchange in UVB group.

KEGG enrichment showed that the DEPs from BC and UVB groups were also significantly mapped in the phenylpropanoids biosynthesis pathway. Since phenylalanine and tyrosine were two important precursors of phenylpropanoids, we then focused on the phenylalanine and tyrosine biosynthesis pathway. The results showed that the abundance of proteins involved in phenylalanine and tyrosine biosynthesis was increased under either BC or UVB stress (Figure 9A). For instance, 3-deoxy-7-phosphoheptulonate synthase (DHS, EC: 4.1.2.15), which is the first committed enzyme of the shikimate pathway, was increased in the BC and UVB groups. Moreover, agrogenate/prephenate dehydratase (ADT/PDT, EC: 4.2.1.91; 4.2.1.51), bifunctional aspartate aminotransferase and glutamate/aspartate-prephenate aminotransferase (PPAT, EC: 2.6.1.1; 2.6.1.78, 2.6.1.79), aspartate aminotransferase (AAT, EC: 2.6.1.1), and tyrosine aminotransferase (TAT, EC:2.6.1.5) were all increased in both the BC and UVB groups (Figure 9A).

### 2.7. Proteins Related to Jasmonic Acid Biosynthesis Were Altered in the BC Group

Stress resistance is modulated by various mechanisms, including phytohormone regulation. Proteins related to the biosynthesis of phytohormones, including auxin, salicylic acid (SA), jasmonic acid (JA), and abscisic acid (ABA), were analyzed based on the proteomics data. The results showed that only proteins related to JA biosynthesis were significantly altered in the BC group, which was different from the UVB group (Figure 9B). Two lipoxygenases (LOX25, EC: 1.13.11.12), a hydroperoxide dehydratase (AOS, EC: 4.2.1.92), four 12-oxophytodienoic acid reductases (OPR, EC: 1.3.1.42), and an OPC-8:0 CoA ligase 1 (OPCL1, EC:6.2.1.-) were identified to be differentially increased in the BC group, while only an OPR increased and an LOX25 decreased in the UVB group.

## 3. Discussion

### 3.1. Lignin and Flavonoid Biosynthesis Were Both Enhanced under B. cinerea Infection and UV-B Stress

Through a Venn diagram, more than half of the DEPs were common in the UVB and BC groups. It seems that many of the changes in response to the UVB and BC treatments were similar, although they were two completely different types of stress. Moreover, the similarity was more obvious when we considered the changes in the pathways related to phenylpropanoid biosynthesis. The common DEPs were not only identified in the lignin biosynthesis pathway and flavonoid biosynthesis pathway, but were also systematically increased. It has been reported that plants suppress the accumulation of flavonoids and up-regulate lignin and phytotoxin biosynthesis when facing biotic stress, especially the down-regulation of the CHS [6]. This kind of suppression is observed in *Glehnia littoralis* cells, *Arabidopsis* seedings, *Brassica napus* seedings, etc. [6,20,21]. However, this kind of suppression was not observed in *M. alba* leaves under BC stress. The abundance of DEPs involved in flavonoid and lignin biosynthesis was increased, and the contents of flavonoids in the BC and UVB groups were also increased. The up-regulation of flavonoid biosynthesis under BC stress was also exhibited on a transcript level [22] (Figure S1). However, *B. cinerea* infection and UV-B stress were two different types of stress, and the distinct change in JA biosynthesis showed that there was a difference in the initial steps of the response under these two types of stress. The similar changes observed in flavonoid and lignin biosynthesis suggested that there might be a same node in the responses to *B. cinerea* infection and UV-B stress, which could up-regulate the proteins involved in flavonoid and lignin biosynthesis simultaneously.

### 3.2. Accumulation of Flavonoids Contributes to B. cinerea Infection Resistance and UV-B Resistance in M. alba Leaves

In the UVB and BC groups, phenylpropanoid biosynthesis and flavonoids biosynthesis were in the several top enriched GO and KEGG terms. The results of pathway mapping showed that many of the key enzymes involved in these two pathways were increased after treatments. Moreover, diverse flavonoids were also identified to be differentially accumulated through non-target metabolomic analysis. Although there were DEPs identified in other secondary metabolite biosynthesis pathways, none of them exhibited such a systematic change like phenylpropanoid biosynthesis, especially flavonoid biosynthesis. The systematic activation of flavonoid biosynthesis was observed on a protein level as well as a metabolite level. It has been reported that plants enhance stress tolerance by promoting flavonoid accumulation. The production of Flavonol Glycosides 3 (PFG3), an R2R3-MYB TF, has been reported to regulate flavonoid biosynthesis in *Arabidopsis*. PFG3-deficient mutants (pfg3 and pfg3-d1) exhibited an impaired accumulation of flavonoids and were more sensitive to osmotic and drought stresses, which indicated that the suppressed tolerance caused by the deficiency of flavonoid biosynthesis could not be complemented by other processes [23]. The polymethoxyflavones (nobiletin, sinensetin, heptamethoxyflavone, and tangeretin) from tangelo Nova fruit could act as natural and in situ inhibitors against a *Phytophthora citrophthora* attack in fruit. In addition, the fruits treated with 6-benzylaminopurine could increase the level of polymethoxyflavone and consequently show greater resistance to an attack by *P. citrophthora* [24]. So, the accumulation of flavonoids could contribute to the resistance of different types of stresses as well, regardless of whether the stress is biotic or abiotic.

Besides the benefit of flavonoid itself, the accumulated flavonoids could serve as a precursor for the production of complex secondary metabolites. Methylcyclohexene motifs, which are accessible via Diels-Alder reactions, are common in Moraceae natural products [25]. Flavonoids are an indispensable precursor of the biosynthesis of methylcyclohexene motifs products in *M. alba* leaves, including kuwanon H, kuwanon J, kuwanol E, guangsangon E, chalcomoracin, etc. These complex compounds showed different activities, including antibacterial and photoprotection. Kuwanon G is an inhibitor of protein tyrosine phosphatases A and B, and showed an inhibition of *Mycobacterium tuberculosis* growth by 61% [26]. Kuwanon G and kuwanon H displayed high efficiency in killing diverse methicillin-resistant Staphylococcus aureus isolates [27]. Kuwanon L could inhibit HIV-1 integrase catalytic activity and HIV-1 replication in cell culture. A study on kuwanon O showed that kuwanon O presented inhibitions on UV-A- or H_2_O_2_-induced cellular ROS and the enhanced repair of DNA damage induced by UV-A [28]. Chalcomoracin is capable of scavenging the superoxide anion generated by the xanthine-xanthine oxidase system and inhibiting lipid peroxidation, which strongly indicates its role as a free radical scavenger under UV-B stress [29]. While these activities were mainly confirmed in vitro, the excellent antibacterial and photoprotection activities of those methylcyclohexene motif compounds derived from flavonoid suggested their important role in the resistance to UV-B stress and *B. cinerea* stress. A previous study of Liu et al. showed that the content of chalcomoracin was increased after UVB treatment and BC treatment, which further emphasized its role in stress resistance [22].

### 3.3. Lignin Biosynthesis Contributes to B. cinerea Resistance and UV-B Resistance in M. alba Leaves

Proteomic data, enzyme assays, and qRT-PCR showed that lignin biosynthesis was activated after *B. cinerea* infection and UV-B stress. Lignin is an indispensable polymer in plants which provides the strength for plants to grow tall in gravitropic environments and a hydrophobic lining to facilitate water and nutrient transport in plant vasculature [30]. Apart from the role of lignin in plant development, it is also widely accepted that lignin is a major player in the response to various biotic and abiotic stresses [31]. Lignin can enhance mechanical strength, change the compressibility and porosity of cell walls, and thus form a barrier against pathogen infections and restrict the movement of pathogen [32]. The infection and spreading of *B. cinerea* require the penetration of the host tissues by the hyphae. Under BC stress, activated lignin biosynthesis can benefit *M. alba* leaves through the strengthened cell wall.

It was commonly reported that flavonoids and lignin had excellent UV absorption ability owing to their aromatic structure and the presence of numerous phenolic, ketone, and intramolecular hydrogen bonds [33]. It has been reported that *Arabidopsis* mutants defective in the ability to synthesize sinapate esters in ferulic acid hydroxylase 1 (fah1) are more sensitive to UV-B than the wild-type. And despite its ability to accumulate flavonoids, the ferulic acid hydroxylase mutant fah1 exhibits more physiological injury than either the wild-type or mutants defective in the synthesis of flavonoids in transparent testa 5 (tt5) [34]. *A. thaliana* plants expressing R2R3-MYB TF, ZmMYB31, exhibited a significant decrease in lignin content and a reduction in sinapoyl malate biosynthesis relative to the control plants, but increased the flavonoid biosynthesis relative to the controls. Despite the increase in flavonoids, transgenic *A. thaliana* plants were still highly sensitive to UV radiation compared with the controls, displaying an upward leaf curling phenotype resembling those observed in mutant plants deficient in UV-protectant compounds [35]. These studies showed that the role of lignin biosynthesis in UV-B resistance was indispensable, which could not be complemented by the accumulation of flavonoids.

## 4. Materials and Methods

### 4.1. Materials and Treatments

The seeds of *M. alba* were washed and sown in soil for gemination. The seedlings were then grown at 25 ± 1 °C with a 16 h light/8 h dark cycle (white-light irradiance, 160 μmol m^−2^ s^−1^) for 5–6 weeks. The treatments of UVB and BC were conducted according to Liu et al. [22], with minor modifications. Briefly, the middle 3–4 pieces of leaves were detached and the petioles were wrapped with wet cotton balls for either the UVB or BC treatment. For the control group, leaves were frozen in liquid nitrogen without any treatment, and then stored at −80 °C for further experiment. For the UVB treatment, UV-B radiation was emitted by 4 tubular low-pressure mercury-vapor lamps (TL40w/12RS, 280–315 nm, Philips, Amsterdam, The Netherlands) and the intensity on the leaf surface was 104.37 kJ m^−2^ d^−1^ [36]. The UV Light Meter (Beijing Normal University, Beijing, China) was used for pertinent measurements. The detached leaves were treated with UV-B radiation for 15 min, then transferred to Petri dishes to retain moisture and incubated in darkness for 36 h. After that, the leaves were frozen in liquid nitrogen, then stored at −80 °C for future use. For the BC treatment, each leaf was incubated with mycelium disks (4 × 4 mm diameter) taken from 10-day-old *B. cinerea* grown on potato dextrose agar medium and placed in a covered Petri dish and incubated for 36 h with a 16 h light/8 h dark cycle. After that, the leaves were frozen in liquid nitrogen, then stored at −80 °C for future use. Three biological replications were performed.

### 4.2. Protein Extraction, Purification, and Digestion

Protein extraction, purification, and digestion were performed according to Zhu et al. [37]. Briefly, leaves were ground into powder in liquid nitrogen and then transferred to an acetone solution containing 10% trichloroacetic acid and 0.07% 2-meraptoethanol. After being vortexed, the mixture was sonicated for 5 min. The suspension was incubated at −20 °C for 1 h and vortexed every 15 min. Then, the suspension was centrifugated at 9000× *g*, 4 °C for 20 min. The supernatant was discarded, and the pellet was washed twice with 0.07% 2-mercaptoethanol in acetone. The pellet was dried using a Speed-Vacconcentrator and resuspended in lysis solution (7 M urea, 2 M thiourea, 5% CHAPS and 2 mM tributylphosphine) by vortex at room temperature for 1 h. The supernatant was collected after centrifugation at 13,000× *g* for 20 min. Protein concentration was determined using a Bradford assay with bovine serum albumin as the standard. The proteins (100 μg) were purified with methanol and chloroform to remove impurities from the samples. After that, the protein was reduced with 50 mM dithiothreitol at 56 °C for 1 h and alkylated with 50 mM iodoacetamide in the dark at 37 °C for 1 h. Then, the protein was digested with trypsin at 1:100 enzyme/protein ratio (*w*/*w*) at 37 °C overnight. The obtained peptides were desalted with ZipTip (Merck Millipore, Billerica, MA, USA), lyophilized, and then dissolved in 0.1% formic acid for nanoflow liquid chromatography-mass spectrometry.

### 4.3. Nanoflow Liquid Chromatography-Tandem Mass Spectrometry Analysis of Proteomics

The instrument method was carried out according to Liu et al. [38]. In brief, the peptides were analyzed on an Easy-nLC 1200 system coupled with an Orbitrap Exploris 480 mass spectrometer (Thermo Scientific Inc., San Jose, CA, USA). The peptides in 0.1% formic acid were loaded onto an Acclaim PepMap 100 C18 column (250 mm × 75 µm, 2 µm-C18) and then eluted with a linear acetonitrile gradient (2–35%) at a flow rate of 300 nL min^−1^ for 120 min.

### 4.4. Protein Identification and Data Processing

The raw data were analyzed using Proteome Discoverer software (version 2.4, Thermo Fisher Scientific Waltham, MA, USA), and tandem mass spectra were searched against Morus notabilis proteins in the UniProt database (Taxon ID: 981085, 26,910 entries, downloaded on 12 October 2021). The oxidation of methionine was set as a variable modification, and the carbamidomethylation of cysteine was set as a fixed modification. The mass tolerance values of MS and MS/MS were set to 10 ppm and 0.2 Da, respectively. The enzyme digestion method was set as trypsinization. Proteins with unique peptides of less than 2 were excluded. Protein abundances in the UVB and BC groups were compared with those in the control group, and proteins with an adjust *p*-value of less than 0.05 were regarded as differentially expressed proteins (DEPs).

### 4.5. GO and KEGG Enrichment of DEPs

The identified proteins were annotated by the eggNOG database [39]. Gene Ontology (GO) and Kyoto Encyclopedia of Genes and Genomes (KEGG) enrichment were performed using TBtools v1.09 based on the annotation of the eggNOG database [40,41,42,43].

### 4.6. Metabolite Extraction from M. alba Leaves and Untargeted Metabolomic Analyses

Metabolites were extracted according to the method of Zhong et al. [44]. In brief, 10 mg of *M. alba* leaf powder was extracted with 0.5 mL 70% methanol for three times. The supernatants were combined, lyophilized, and reconstituted in 50 µL of distilled water. Untargeted metabolomics was conducted using a Vanquish Horizon UPLC system (Thermo Scientific, Les Ulis, France) coupled with a Thermo Scientific Orbitrap Exploris 480 mass spectrometer. A reverse-phase C18 column (Waters, BEH C18, 100 × 2.1 mm, 1.7 µm) was used for metabolite separation with 0.1% formic acid in water as solvent A and 0.1% formic acid in acetonitrile as solvent B. The flow rate was 0.4 mL min^−1^. The LC gradient started at 0.1% solvent B for 0.5 min, then a linear gradient from 0.1% B to 40% B over 21 min, then a linear gradient from 40% B to 90% B over 2 min, held at 90% B for 2 min, and then return to 0.1% B.

### 4.7. Metabolomic Data Processing

The raw data of metabolomic analysis were processed using Compound Discoverer 3.2 (Thermo Fisher Scientific Waltham, MA, USA). Metabolite annotation was performed by searching the raw data against the mzCloud, mzVault, KEGG, AraCyc, BioCyc, Plant Cyc, and ChemSpider databases with a mass tolerance of 10 ppm. The identified metabolites were assigned as “level 2-identified compounds” because of MS/MS database matching [45]. The contents of the metabolites in the UVB and BC groups were compared with those in the control group, and the metabolites with an adjust *p*-value of less than 0.05 were regarded as differential abundance metabolites (DAM).

### 4.8. Quantitative Real-Time PCR

RNA extraction and qRT-PCR analysis were performed according to Liu et al. [46]. The primer information used in this study is shown in Table S1. The relative gene expression and fold change were calculated with the 2^−ΔΔCt^ method.

### 4.9. Analysis of Physiological Changes

The enzyme activities of Cinnamyl-alcohol dehydrogenase (CAD) and L-phenylalanine ammonialyase (PAL) were measured using the CAD assay kit (Leagene, Beijing, China) and PAL assay kit (Leagene, Beijing, China) according to the manuals. The contents of total flavonoids were measured using the total flavonoids content assay kit (BioSharp, Hefei, China).

## 5. Conclusions

In this study, we compared the response to *B. cinerea* stress and UV-B stress in *M. alba* leaves. Proteomic analysis showed that flavonoid biosynthesis and lignin biosynthesis were both activated under two types of stress. The activation was further confirmed by enzyme activity assays and metabolite analysis. Phenylalanine and tyrosine biosynthesis, which is the upstream of flavonoid biosynthesis and lignin biosynthesis, were also up-regulated. In addition, the biosynthesis of different phytohormones was also evaluated, and a distinction was found in the JA biosynthesis between *B. cinerea* infection and UV-B stress. These results indicated that although *B. cinerea* infection and UV-B radiation are two different types of stress and may activate different signaling pathways, both of them could finally up-regulate flavonoid biosynthesis and lignin biosynthesis. Moreover, these two branches of phenylpropanoid metabolism contributed to the resistance to both types of stress in *M. alba*.

## Data Availability

The metabolomics raw data files are freely available via scientific data repository Zenodo.org. https://doi.org/10.5281/zenodo.8284018 (accessed on 5 August 2023).

## Supplementary Materials

The following supporting information can be downloaded at: https://www.mdpi.com/article/10.3390/plants12183265/s1, Figure S1: DEGs in flavonoid biosynthesis; Table S1: Primers used in qRT-PCR.
