# Supplementary material for: Integrative Omic Analysis Reveals the Dynamic Change in Phenylpropanoid Metabolism in Morus alba under Different Stress
Source: Plants (Basel). 2023 Sep 14;12(18):3265. doi: 10.3390/plants12183265 (PMC10537046; doi:10.3390/plants12183265)
Supplement: Supplementary file 1 [file plants-12-03265-s001.zip › plants-2603486-supplementary.pdf]

Table S1 Primers used in qRT-PCR

**Primer**	**Sequence**
COMT-F	GTCGACCGTGCTGTCTATGT
COMT-R	GCAATTGAGCACAGAGTGGC
β-actin-F	AGGGGAAGCTGGCTTATGTT
β-actin-R	CGGGCAGCTCATAGTTCTTC

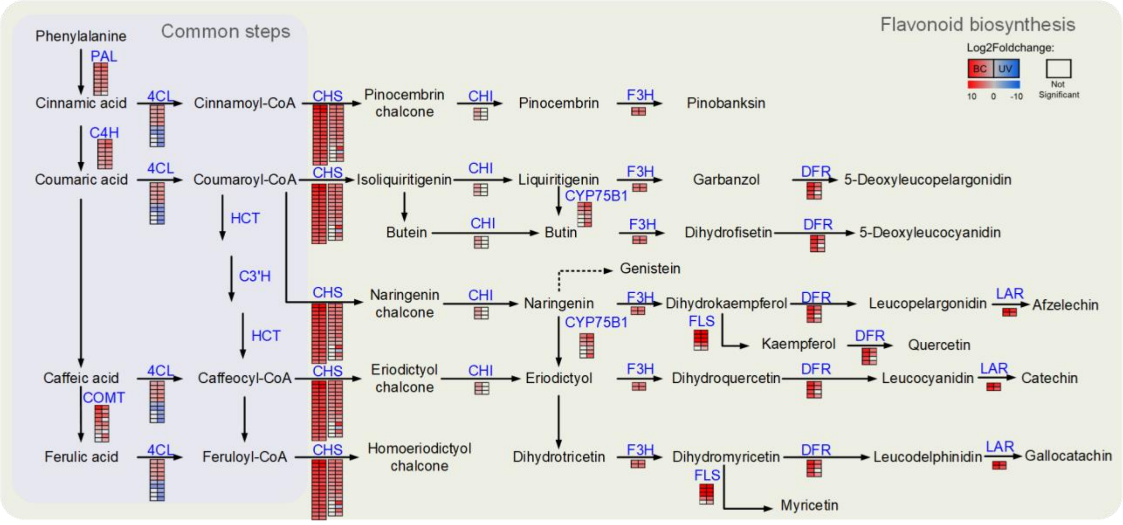

Figure S1 DEGs in flavonoid biosynthesis. Data was extracted from published transcriptome study of M. alba leaves [20]. Each line of squares represented an RNA. Filled color represented Log2Foldchange of RNA. Left column represented Log2Foldchange in BC group, and right column represented Log2Foldchange in UVB group.
